# Deletion of Slc6a14 reduces cancer growth and metastatic spread and improves survival in KPC mouse model of spontaneous pancreatic cancer

**DOI:** 10.1042/BCJ20210855

**Published:** 2022-03-16

**Authors:** Bradley K. Schniers, Mitchell S. Wachtel, Meenu Sharma, Ksenija Korac, Devaraja Rajasekaran, Shengping Yang, Tyler Sniegowski, Vadivel Ganapathy, Yangzom D. Bhutia

**Affiliations:** 1 Department of Cell Biology and Biochemistry, Texas Tech University Health Sciences Center, Lubbock, TX 79430, U.S.A.; 2 Department of Surgical Pathology, Texas Tech University Health Sciences Center, Lubbock, TX 79430, U.S.A.; 3 Pennington Biomedical Research Center, Louisiana State University, Baton Rouge, LA 70808, U.S.A.

**Keywords:** ascites fluid, KPC, metastasis, pancreatic cancer, SLC6A14, survival

## Abstract

Pancreatic ductal adenocarcinoma (PDAC) is lethal. There is a dire need for better therapeutic targets. Cancer cells have increased demand for sugars, amino acids, and lipids and therefore up-regulate various nutrient transporters to meet this demand. In PDAC, SLC6A14 (an amino acid transporter (AAT)) is up-regulated, affecting overall patient survival. Previously we have shown using *in vitro* cell culture models and *in vivo* xenograft mouse models that pharmacological inhibition of SLC6A14 with α-methyl-l-tryptophan (α-MLT) attenuates PDAC growth. Mechanistically, blockade of SLC6A14-mediated amino acid transport with α-MLT leads to amino acid deprivation, eventually inhibiting mTORC1 signaling pathway, in tumor cells. Here, we report on the effect of Slc6a14 deletion on various parameters of PDAC in KPC mice, a model for spontaneous PDAC. Pancreatic tumors in KPC mice show evidence of Slc6a14 up-regulation. Deletion of *Slc6a14* in this mouse attenuates PDAC growth, decreases the metastatic spread of the tumor, reduces ascites fluid accumulation, and improves overall survival. At the molecular level, we show lower proliferation index and reduced desmoplastic reaction following *Slc6a14* deletion. Furthermore, we find that deletion of *Slc6a14* does not lead to compensatory up-regulation in any of the other amino transporters. In fact, some of the AATs are actually down-regulated in response to Slc6a14 deletion, most likely related to altered mTORC1 signaling. Taken together, these results underscore the positive role SLC6A14 plays in PDAC growth and metastasis. Therefore, SLC6A14 is a viable drug target for the treatment of PDAC and also for any other cancer that overexpresses this transporter.

## Introduction

Pancreatic ductal adenocarcinoma (PDAC) is lethal. There is a dire need for more efficient treatments to improve survival outcomes [1–3]. Cancer cells have a rapid proliferation capacity and therefore exhibit increased demand for nutrients such as sugars, amino acids, lipids, and vitamins. To acquire these nutrients, cancer cells significantly up-regulate several nutrient transporters for their benefit. Pharmacological targeting of these nutrient transporters to ‘starve’ the tumor cells to death seems a logical and viable therapeutic strategy.

We have found SLC6A14 to be significantly up-regulated in PDAC [4]. SLC6A14, also known as ATB^0,+^, is an amino acid transporter (AAT) energized by three driving forces: a Na^+^ gradient, a Cl^−^ gradient, and a membrane potential. With a broad substrate selectivity, it can transport 18 of the 20 amino acids, which includes all essential amino acids, and primarily mediates the influx of its substrates into cells because of the high magnitude of the combined driving forces [5–8]. Apart from PDAC, SLC6A14 is also significantly up-regulated in colorectal cancer [9,10], estrogen receptor (ER)-positive breast cancer [11,12], and cervical cancer [13]. Using *in vitro* cell line models as well as *in vivo* xenograft mouse models, we have shown that pharmacological inhibition of SLC6A14-mediated amino acid transport with α-methyl-l-tryptophan (α-MLT) attenuates PDAC growth [4]. Mechanistically, we have shown that blockade of SLC6A14 essentially deprives the cancer cells of amino acids, which in turn inhibits mTORC1 signaling pathway, affecting overall protein translation [4]. Likewise, literature evidence shows similar findings in colorectal cancer and ER-positive breast cancer, further establishing SLC6A14 as a tumor promoter and a potential drug target for cancer therapy [10,11].

Genetically engineered mice are vital to understand tumorigenesis and find answers to questions that other systems cannot address. Though cell culture and xenograft mouse models have led to valuable insights into the role of SLC6A14 in PDAC, these systems do not recapitulate the disease in humans as these studies were done in the absence of native tissue microenvironment and a competent immune system. Therefore, in this study, we set out to evaluate the role of Slc6a14 in a spontaneous mouse model of PDAC. Specifically, we wanted to determine the consequences of Slc6a14 deletion in *LSL-Kras^G12D/+^; LSL-p53^R172H/+^; Pdx-1 Cre* (KPC) mice in terms of PDAC growth, metastatic spread of the tumor, ascites fluid accumulation, and overall survival. KPC mouse is a widely accepted spontaneous model of PDAC wherein oncogenic Kras^G12D^ and Trp53^R172H^ have been genetically introduced, in conjunction with a pancreas-specific Cre-recombinase [14]. The incorporated mutations i.e. Kras^G12D^ and Trp53^R172H^, an ortholog of the human gene, are commonly found in human PDAC. In fact, the mutation frequency of KRAS proto-oncogene is >90% and that of the TP53 tumor suppressor gene is >75% in all human PDACs. Hence, it is not surprising that the KPC model recapitulates many of the salient clinical (e.g. ascites development, bowel and biliary obstruction, and cachexia) and histopathological features (e.g. poor vascularity, fibrosis, and metastasis) of the human disease. Therefore, results obtained from these mice will be a valuable preclinical data.

In the present study, we first generated KPC mice in both *Slc6a14^+/+^* (KPC) and *Slc6a14*^−/−^ background (KPCS). With these mice, we show that deletion of Slc6a14 in KPC mice significantly improved their overall survival outcome. Though the KPCS mice ultimately succumbed to the disease, this took a much longer time than in KPC mice. KPCS mice also showed a much-reduced metastatic spread of the tumor and a markedly reduced ascites fluid accumulation. This represents the first report on the essential role of the AAT Slc6a14 in PDAC growth and metastasis using an immunocompetent spontaneous mouse model and underscores the potential of this transporter as a viable drug target for PDAC therapy.

## Results and discussion

### KPC mice develop highly invasive and metastatic PDAC with up-regulated Slc6a14

**Figure 1.**
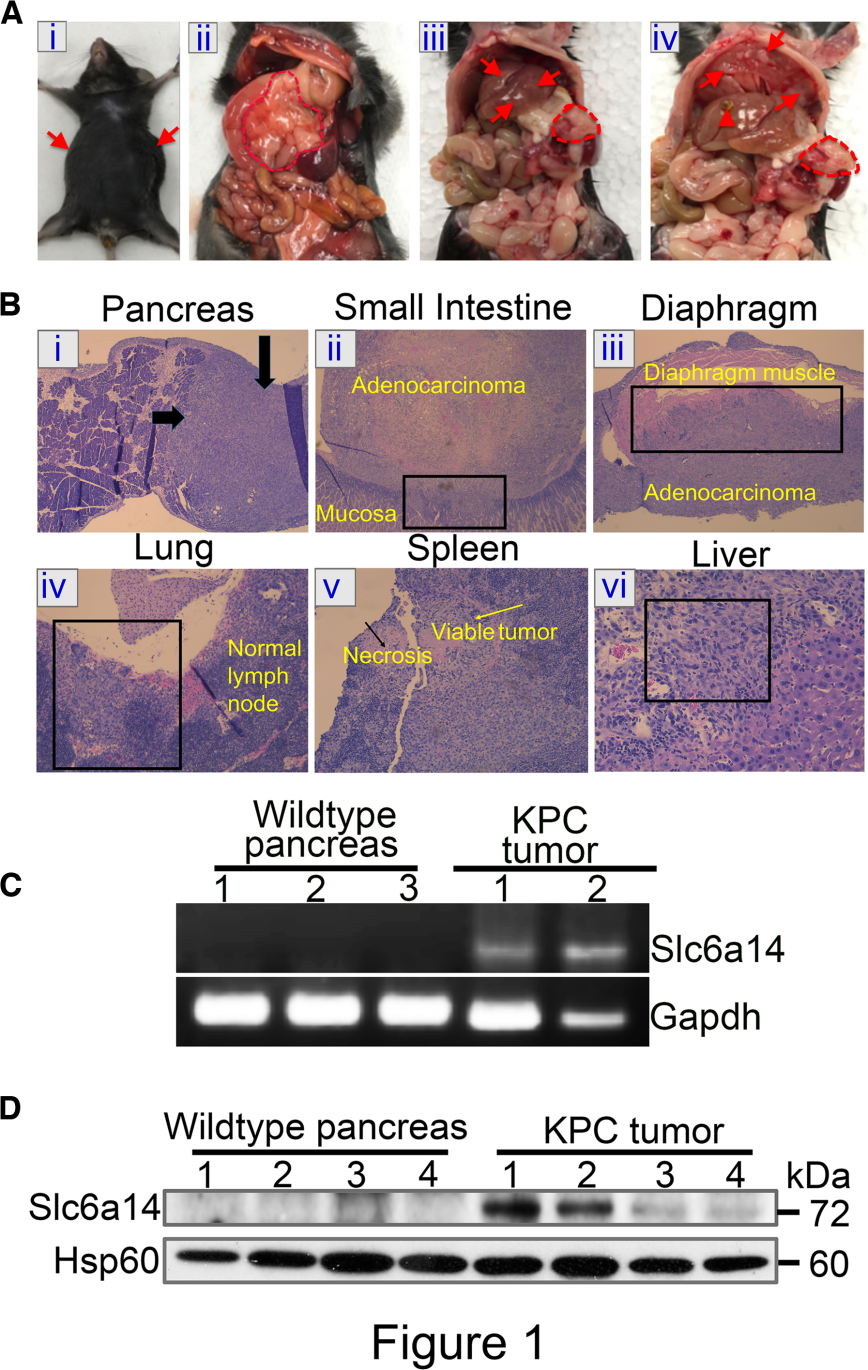
KPC mice develop highly invasive and metastatic PDAC with up-regulated Slc6a14. Gross pathological images of a representative KPC mouse showing abdominal distension due to malignant ascites (**A**-i), primary tumor in the head of pancreas (red outline) (**A**-ii), pancreas tumor (red circle) and liver metastasis (red arrows) (**A**-iii), and metastatic lesions on the diaphragm (red arrows), distended gall bladder (arrow head), and pancreas tumor (red circle) (**A**-iv). Histopathological images of a representative KPC mouse showing pancreas with tumor on the right side indicated by thick arrows (**B**-i), small intestine showing tumor invading through the wall into the mucosa of the small intestine (**B**-ii), tumor invading the diaphragmatic muscle (**B**-iii), enlarged lymph node near the lung, which at higher power shows metastatic tumor nests beneath its capsule (**B**-iv), spleen with partly necrotic tumor with central necrosis (**B**-v), and metastatic extension to the liver (**B**-vi). RT-PCR showing up-regulation of Slc6a14 mRNA expression in tumor pancreas from KPC mice compared with normal pancreas from wild-type mice. Gapdh was used as an internal control (**C**). Western blot showing up-regulation of Slc6a14 protein in tumor pancreas from KPC mice compared with normal pancreas from wild-type mice. Hsp60 was used as an endogenous control (**D**).

KPC mouse is a well-accepted animal model for preclinical studies in the field of PDAC as it exactly phenocopies the disease process in humans. Since Kras^G12D^, coupled with Trp53^R172H^, has been shown to lead to PanIN lesions coupled with aggressive PDAC progression, we based the KPC model used on previously published evidence [14]. To test whether deletion of Slc6a14 in KPC mice will improve their overall survival outcome, we first established the KPC triple transgenic mice in our laboratory. Based on the literature evidence, KPC mice develop full blown PDAC in about 3–5 months, characterized by cachexia, abdominal distension, and median survival of approximately 5 months [14]. To test whether the KPC mice in our laboratory exhibited similar phenotypes, we carefully monitored them for these salient features. It was interesting to observe that all KPC mice established in our facility exhibited similar phenotypes and disease pattern. These included cachexia, distended abdomen, and death within 48–72 h with the onset of these symptoms (Figure 1A). Upon sacrifice, the abdominal cavity contained 5–7 ml of hemorrhagic ascitic fluid. Upon further examination during necropsy, mice were found to typically have a large fibrotic pancreatic mass, metastatic foci in the liver and diaphragm (Figure 1A; i–iv). Following histopathological analysis, the majority of KPC mice were found to have invasive ductal carcinoma of the pancreas followed by the metastatic spread of the tumor to adjacent organs such as the small intestine, diaphragm, lung, spleen, and liver (Figure 1B; i–vi). Having established the KPC model in our facility, our next aim was to confirm the up-regulation of Slc6a14 in PDAC seen in this mouse model as we have seen in human PDAC and human pancreatic cancer cell lines [4]. For this, real-time polymerase chain reaction (PCR) was performed to monitor Slc6a14 mRNA expression in three normal pancreas from wild-type mice, and two tumor pancreas from KPC mice. Interestingly, the normal pancreas did not express Slc6a14 whereas the tumor pancreas showed a significant increase in Slc6a14 mRNA expression (Figure 1C). The up-regulation was also true at the protein level as demonstrated by the western blotting (Figure 1D). This allows us to use the KPC mice as a model to evaluate the role of Slc6a14 in PDAC. Though it is already established that SLC6A14 is up-regulated in many cancer types like the cervical cancer, colorectal cancer, and ER-positive breast cancer; however, the fact that the maximum up-regulation and a significant correlation to patient survival are evident only in PDAC makes this a unique drug target, particularly for this cancer type.

### Deletion of Slc6a14 improves the overall survival in KPC mice

**Figure 2.**
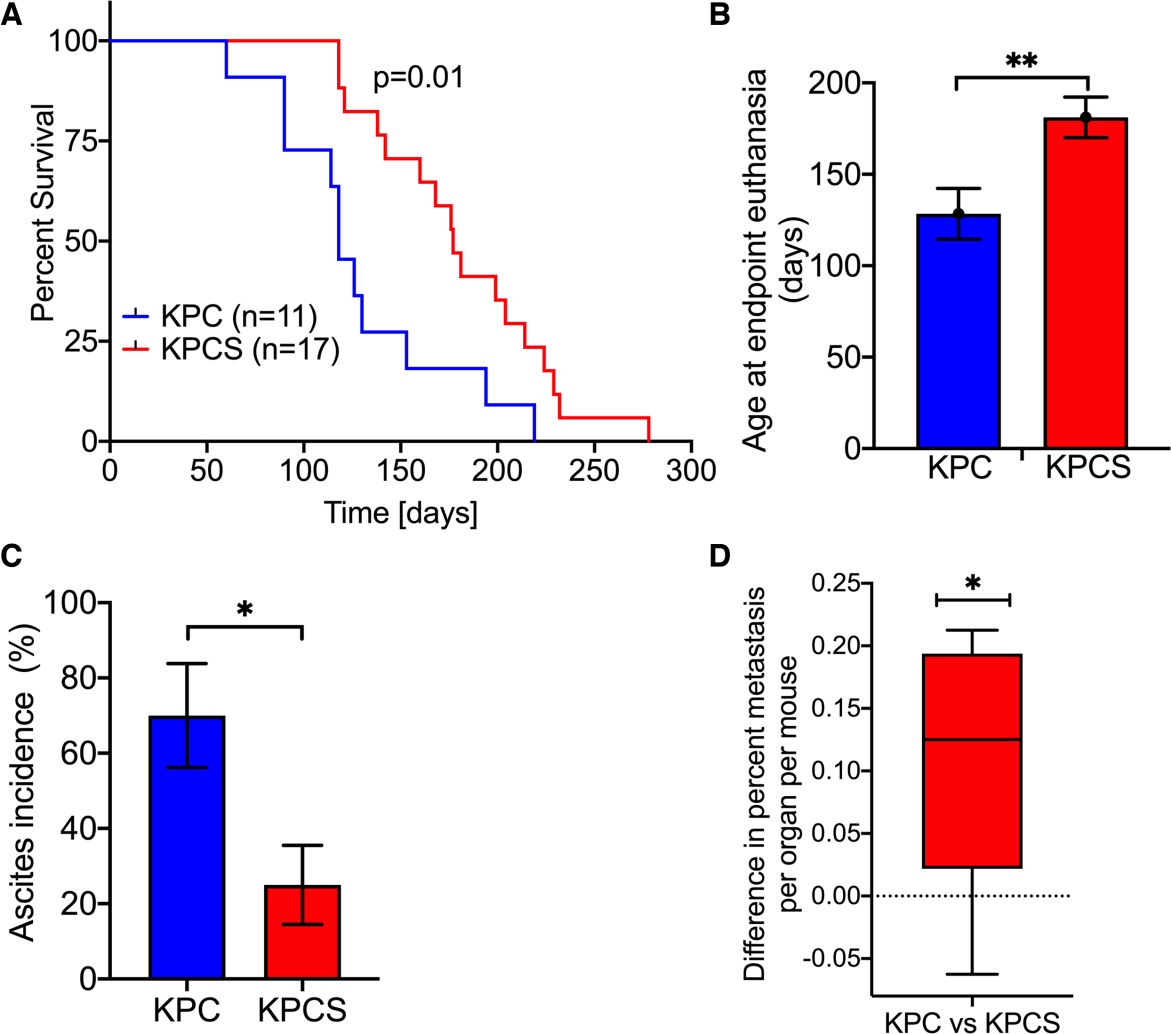
Deletion of Slc6a14 improves several parameters of PDAC in KPC mice. (**A**) Kaplan–Meier curve showing survival outcome between KPC and KPCS mice. (**B**) Bar diagram showing age at endpoint euthanasia in KPC and KPCS mice. (**C**) Bar diagram showing ascites incidence in KPC and KPCS mice. (**D**) Box plot showing the difference in percent metastasis per organ per mouse between the KPC vs the KPCS mice. Data are given as mean ± SEM. *P < 0.05, **P < 0.01.

To test whether deletion of Slc6a14 in KPC mice will improve their overall survival, we first generated the KPC mice (*n *= 11) with *Slc6a14^+/+^* background and the KPCS mice (*n *= 17) with *Slc6a14*^−/−^ background and followed them for their survival. Mice were killed when they reached the endpoint criteria (abdominal distention, cachexia) as stipulated by the Institutional Animal Care and Use Committee (IACUC). Clearly, deletion of Slc6a14 significantly prolonged the survival outcome in KPC mice (Figure 2A). While the KPC mice had a shortened median survival of about 3.9 months, KPCS mice on the other hand had an enhanced median survival of about 5.9 months, a 2-month increase in medial survival as a consequence of Slc6a14 deletion. At the endpoint mandated for euthanasia by IACUC, the age of the KPC mice was 128.4 ± 13.9 days (*n *= 11) and the corresponding value for KPCS mice was 181.1 ± 11.1 days (*n *= 17) (Figure 2B). This represents a 41% increase in the maximal lifespan as a result of Slc6a14 deletion. Though the 2-month difference in the median survival does not seem quite a lot in mouse lifespan, this difference corresponds to about 6.6 human years [15]. This is a significant improvement in median survival and in maximal lifespan. There was also a dramatic decrease in the incidence of ascites in KPCS mice (25%) compared with KPC mice (70%) (Figure 2C). A majority of the KPC mice (64%) developed invasive ductal carcinoma of the pancreas and metastasis to the liver (40%), lung (40%), diaphragm (40%), spleen (10%), stomach (30%), and intestine (20%). Metastasis was not seen in the kidneys and the adrenals. The remaining KPC mice either developed lymphoma (18%) or sarcomatoid carcinoma of the pancreas (9%) (Table 1). Likewise, a majority of the KPCS mice developed invasive ductal carcinoma of the pancreas (65%) and metastasis to the liver (31%), lung (25%), diaphragm (19%), stomach (13%), and kidneys (6%). No metastasis was seen in the spleen, intestine, and the adrenals. The remaining KPCS mice developed sarcomatoid carcinoma (6%), high-grade gluteal sarcoma (6%), or had no carcinoma (12%) (Table 2). But it has to be noted here that the metastatic spread of the tumor was evaluated at the endpoint, meaning that the values observed for metastatic incidence in different organs are at a much younger age in KPC mice than in KPCS mice. Moreover, comparing the organ-level metastasis between the two groups of mice, it was interesting to observe that the KPC mice had 12.5% more metastatic counts than the KPCS mice (Figure 2D).

**Table 1.** Clinical spectrum of disease in *LSL-Kras^G12D/+^; LSL-Trp53^R172H/+^; Pdx-1-Cre* (KPC) mice

Sr. no.	Age (days)	Sex/animal ID	Histological diagnosis	Metastasis								Ascites
				Liver	Lung	Diaphragm	Spleen	Stomach	Intestine	Kidneys	Adrenals	
1	60	F2	Lymphoma	N	N	N	N	N	N	N	N	N
2	126	F3	Invasive ductal carcinoma (moderately differentiated)	Y	Y	Y	N	N	N	N	N	Y
3	153	F4	Invasive ductal carcinoma (moderately differentiated)	Y	N	Y	N	N	Y	N	N	Y
4	219	F6	Invasive ductal carcinoma (moderately to poorly differentiated)	Y	Y	N	N	N	N	N	N	Y
5	118	F7	Invasive ductal carcinoma (moderately to poorly differentiated)	N	N	N	N	Y	N	N	N	N
6	90	F10	Sarcomatoid carcinoma of pancreas	N	N	N	N	N	N	N	N	Y
7	90	M4	Malignant lymphoma	N	N	N	N	N	N	N	N	Y
8	114	M9	Invasive ductal carcinoma (poorly differentiated)	Y	Y	Y	Y	Y	Y	N	N	Y
9	118	F6	Tissues not evaluated due to necrosis. Had tumor in pancreas	*	*	*	*	*	*	*	*	N
10	194	M4	Invasive ductal carcinoma	N	Y	Y	N	Y	N	N	N	Y
11	130	M3	Pancreas cystic lesion	N	N	N	N	N	N	N	N	N
Total			7/11	4/10	4/10	4/10	1/10	3/10	2/10	0/10	0/10	7/10

Y, yes; N, no; *, tissue not evaluated.

**Table 2.** Clinical spectrum of disease in *LSL-Kras^G12D/+^; LSL-Trp53^R172H/+^; Pdx-1-Cre; Slc6a14*^−/−^ (KPCS) mice

Sr. no.	Age (days)	Sex/animal ID	Histological diagnosis	Metastasis								Ascites
				Liver	Lung	Diaphragm	Spleen	Stomach	Intestine	Kidneys	Adrenals	
1	118	F7	Invasive ductal carcinoma (moderately differentiated)	N	N	N	N	N	N	N	N	N
2	181	M1	Invasive ductal carcinoma (well differentiated)	N	Y	N	N	N	N	N	N	N
3	229	M2	Invasive ductal carcinoma (poorly differentiated)	Y	N	N	N	N	N	N	N	N
4	199	F1	Invasive ductal carcinoma (moderate to poorly differentiated)	N	Y	Y	N	N	N	Y	N	Y
5	232	F1	Invasive ductal carcinoma (moderate to poorly differentiated)	N	N	Y	N	Y	N	N	N	Y
6	204	F2	Invasive ductal carcinoma (well differentiated)	Y	N	N	N	N	N	N	N	N
7	118	F7	Tissues not evaluated due to necrosis. No apparent tumor	*	*	*	*	*	*	*	*	*
8	121	M1	No carcinoma identified	N	N	N	N	N	N	N	N	N
9	160	M3	High-grade sarcoma of the gluteal region	Y	Y	N	N	N	N	N	N	N
10	176	M4	Sarcomatoid carcinoma	N	N	N	N	N	N	N	N	N
11	142	M5	Invasive ductal carcinoma (poorly differentiated)	N	N	N	N	Y	N	N	N	N
12	214	M7	Invasive ductal carcinoma (mostly poorly differentiated)	N	N	Y	N	N	N	N	N	Y
13	177	F1	Positive for malignancy; lesional tissue has spindle cell morphology	N	N	N	N	N	N	N	N	Y
14	224	F2	Invasive ductal carcinoma	Y	N	N	N	N	N	N	N	N
15	138	M4	Invasive ductal carcinoma	Y	Y	N	N	N	N	N	N	N
16	168	F7	Invasive ductal carcinoma (moderately differentiated)	N	N	N	N	N	N	N	N	N
17	278	F2	No carcinoma identified	N	N	N	N	N	N	N	N	N
Total			11/17	5/16	4/16	3/16	0/16	2/16	0/16	1/16	0/16	4/16

Y, yes; N, no; *, tissue not evaluated.

### Deletion of Slc6a14 reduced the proliferation capacity and the desmoplastic reaction in KPC mice

**Figure 3.**
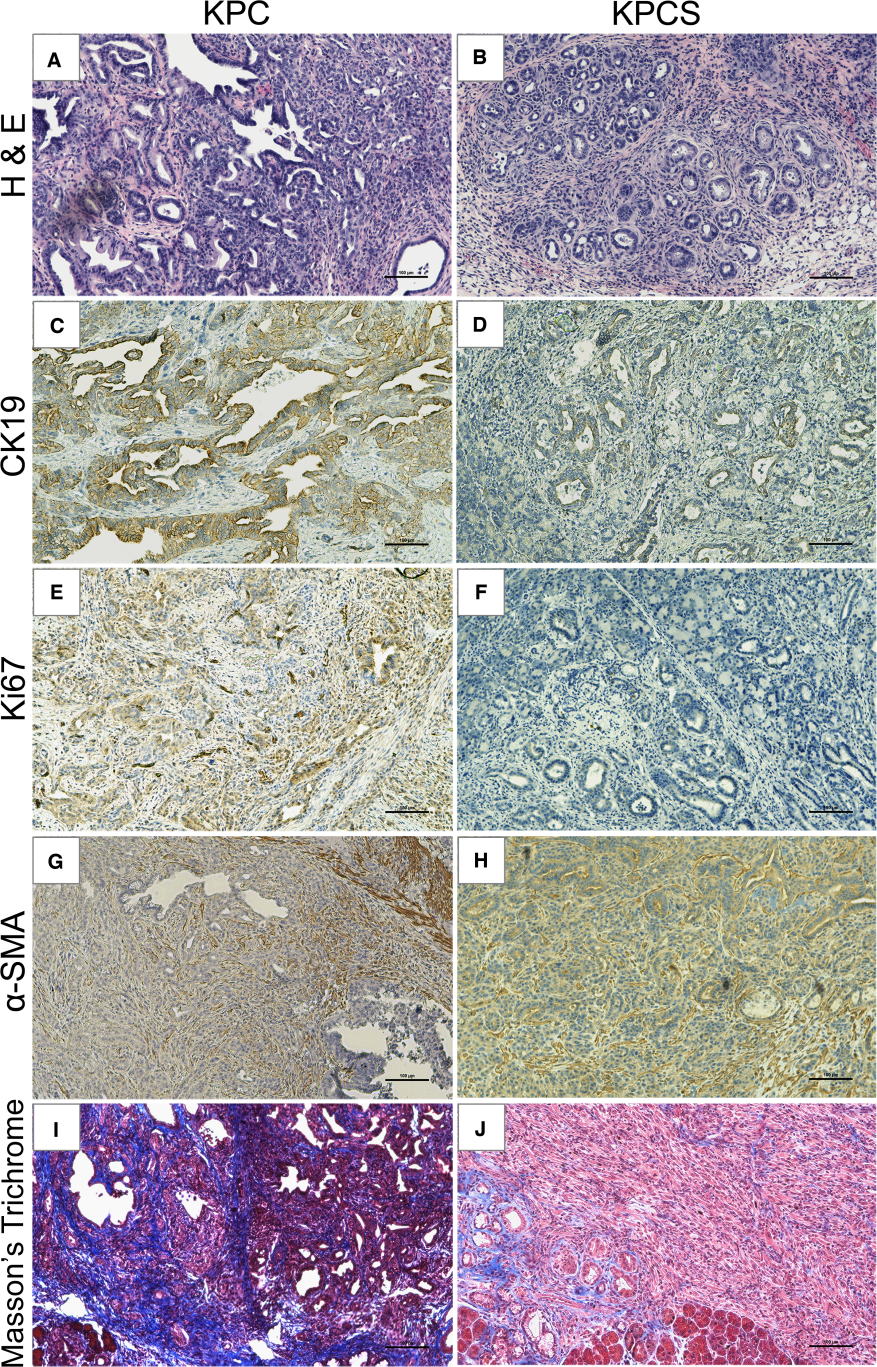
Deletion of Slc6a14 reduces the proliferation capacity and the desmoplastic reaction in the KPC mice. Histopathological staining in KPC and KPCS pancreatic tumor sections (**A**,**B**); immunohistochemical analysis showing staining of CK19 (**C**,**D**), Ki67 (**E**,**F**), α-SMA (**G**,**H**), and Masson's Trichrome (**I**,**J**) in KPC and KPCS pancreatic tumor sections. Scale bar: 100 μm.

We then examined the molecular differences between the KPC and the KPCS tumor sections. Since malignant PDAC is characterized by invasiveness, dedifferentiation, desmoplastic reaction and high proliferation capacity, we performed immunohistochemistry with KPC and KPCS tumor sections and checked the expression status of cytokeratin 19 (CK19), Ki67, alpha-smooth muscle actin (α-SMA), and Masson's Trichrome staining. CK19 is a ductal marker and is used as a marker for adenocarcinoma in the pancreas, with the level of staining indicative of tumor burden [16]. In fact, CK19 is used as a prognostic biomarker in neuroendocrine tumors, with higher levels of CK19 being correlated with poor prognosis. Our results clearly showed that CK19 was expressed at substantially higher levels in KPC tumor sections as opposed to KPCS tumor sections (Figure 3C,D). Next, Ki67 is a proliferation marker, with high levels of Ki67 indicating a significant increase in cell growth and proliferation. It was interesting to note that Ki67 staining was also significantly higher in KPC tumor sections when compared with KPCS tumor sections (Figure 3E,F). Pancreatic cancer in KPC mice is highly aggressive and characterized by the desmoplastic stroma that could be analyzed by α-SMA staining and also by Masson's trichrome that stains collagen. The desmoplastic stroma is unique in PDAC and constitutes up to 80–85% of the tumor bulk. Amongst the cellular components in the tumor microenvironment (TME), pancreatic stellate cells (PSCs) contribute to PDAC desmoplasia. PSCs are the vitamin A storing cells when in the quiescent stage but upon activation, they transdifferentiate into myofibroblasts and produce extracellular matrix (ECM) components mainly collagen, fibronectin, and laminin that contribute to the tumor desmoplastic reaction [17]. Activated PSCs express α-SMA. Our results clearly showed that KPC tumor sections had an intense staining for α-SMA as well as for Masson's trichrome compared with the KPCS sections (Figure 3G–J). Taken together, these results suggest that Slc6a14 deletion leads to a significant reduction in PDAC progression in KPC mice, implying the critical role of SLC6A14 in PDAC.

The fact that SLC6A14 has a broad substrate selectivity explains as to why deleting this transporter in KPC mice would attenuate tumor growth and improve survival outcome. The substrates of SLC6A14 play an obligatory role in cancer cell metabolism and pro-growth cellular signaling: glutamine in glutaminolysis, serine, glycine and methionine in one-carbon metabolism and purine/pyrimidine synthesis, and leucine and arginine in mTORC1 activation. Recent studies have shown that deficiency of arginine, another substrate for SLC6A14, inhibits the adhesion, invasion, and migration of pancreatic cancer cells by decreasing expression of Snail, Slug, Twist as well as matrix metalloproteinases (MMPs) MMP-1 and MMP-9 [18–20]. Furthermore, as a precursor of nitric oxide (NO), arginine is also responsible for proliferation and metastasis [18–20]. Therefore, it is likely that deletion of SLC6A14 curtails all these essential mechanisms in the cancer cells thereby inhibiting their metastatic potential and reducing the incidence of ascites, thus culminating in slower progression and improved survival of the KPCS mice. Taken together, these data clearly demonstrate the tumor-promoting role of SLC6A14 in PDAC, thus underscoring the therapeutic potential of this transporter as a novel and viable drug target for PDAC.

### Deletion of Slc6a14 does not lead to a compensatory up-regulation of other amino acid transporters in the pancreas

**Figure 4.**
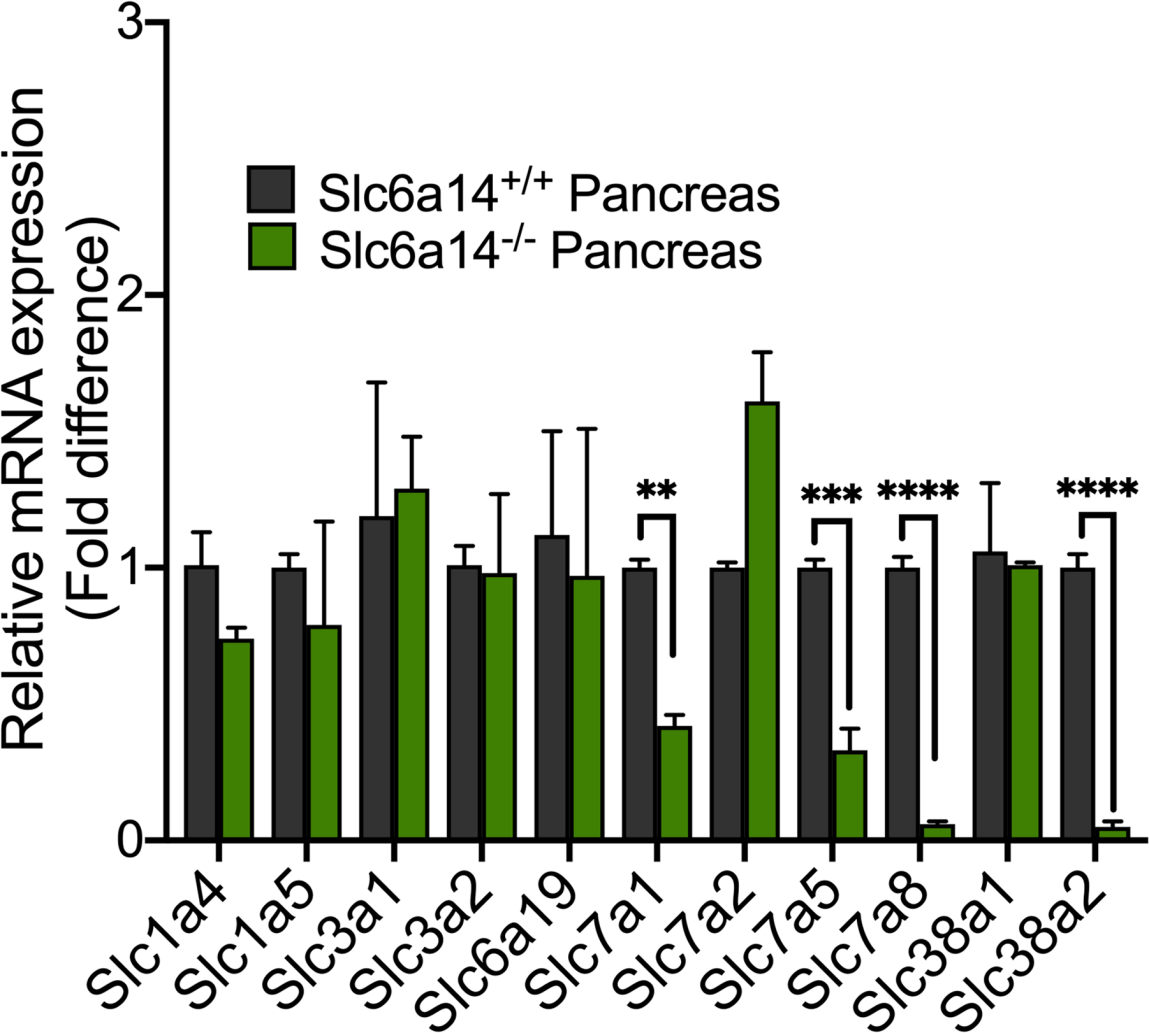
Deletion of Slc6a14 does not lead to a compensatory up-regulation of other amino acid transporters. Real-time PCR showing relative mRNA expression of 12 amino acid transporters in *Slc6a14*^+/+^ (wild-type) pancreas vs *Slc6a14*^−/−^ pancreas. Data are given as mean ± SEM. **P < 0.01, ***P < 0.001, ****P < 0.0001.

While it is important to know that Slc6a14 deletion improves survival in KPC mice, we were curious to find out whether the absence of this transporter would lead to a compensatory up-regulation in other AATs. That said, it has already been published that Slc6a14 knockout mice do not have any overt phenotype and they breed normally [11]. More interestingly, they show no major compensatory changes in the expression of other AATs in the colon and lung, and there were no significant changes in the plasma amino acid profile between wild-type mice and *Slc6a14*-null mice [11]. However, the expression status of other AATs in the pancreas has not yet been investigated in wild-type mice versus *Slc6a14*-null mice. To address this issue, we performed real-time PCR to monitor the mRNA expression of 12 different AATs in the pancreas of wild-type mice and the pancreas of *Slc6a14*-null mice. These experiments showed that most of the transporters examined did not show any difference in expression in response to Slc6a14 deletion (Figure 4). In fact, some of the transporters actually showed a significant decrease in expression in *Slc6a14*-null mice compared with wild-type mice. This included Slc7a1, Slc7a5, Slc7a8, and Slc38a2. Slc7a1, Slc7a5, and Slc7a8 are sodium-independent AATs that transport either cationic amino acids (Slc7a1) or neutral amino acids (Slc7a5 and Slc7a8). Slc38a2 is a sodium-dependent transporter for glutamine and small aliphatic amino acids such as alanine and serine. While there could be a myriad of reasons for their down-regulation, one obvious reason could be the altered mTOR signaling as a result of Slc6a14 deletion. mTORC1 is a sensor of amino acid status within a cell; this signaling pathway gets activated when cells are loaded with amino acids and suppressed when cells are deficient in amino acids. This mode of regulation links cellular amino acid status to protein synthesis because mTOR provides an anabolic signal coupled with the promotion of protein synthesis. Our previous studies have shown that blockade of SLC6A14 function is associated with decreased mTOR signaling [4]. Therefore, the AATs that are down-regulated in *Slc6a14*-null pancreas are the ones whose expression might be under the control of mTOR. This however needs to be investigated. Nonetheless, the absence of up-regulation of any of the AATs examined in *Slc6a14*-null pancreas is an important finding and the down-regulation of some of the AATs is also equally important to understand why SLC6A14 is an ideal therapeutic target for PDAC. Currently, there are few studies analyzing the loss of SLC7A1 in PDAC; however, as an arginine transporter, SLC7A1 could potentially be a source of amino acid nutrients, like other AATs. While SLC7A5 and its relation to cancer has been well established, the role of SLC7A8 to cancer is recently being explored. Previous research has found that PDAC tumors expressing high levels of SLC7A5 have a poor prognostic correlation, while high levels of SLC7A8 correlate to chemoresistance in PDAC [21,22]. Recent studies have also found that inhibition of SLC38A2 deprives PDAC cells of alanine and significantly reduces the proliferation capacity of the cells [23]. These AATs are overexpressed in PDAC and therefore could be targeted for PDAC therapy. Small molecule inhibitors of SLC7A5 and SLC7A8 are already known. However, since the deletion of Slc6a14 resulted in their down-regulation, it is possible that the pharmacological blockade of SLC6A14 will also inhibit the expression of these AATs. If that is true than the need to specifically inhibit these AATs could be avoided. In other words, by simply inhibiting SLC6A14, we could also suppress the expression of any of the above AATs if they are up-regulated concurrently with SLC6A14.

## Conclusion

In conclusion, deletion of Slc6a14 significantly improves overall survival in KPC mice. Slc6a14 deletion is not associated with a compensatory increase in other AATs. More interestingly, Slc6a14 deletion reduced the proliferation capacity and the desmoplastic reaction, which might have contributed to the reduced invasiveness, lower ascites incidence, and thereby prolonged survival in the KPC mice. That said, SLC6A14 also plays a critical role in mTORC1 signaling activation. Therefore, mTORC1 inhibition following Slc6a14 deletion could also be a contributing factor for the observed effects of Slc6a14 deletion on PDAC growth and metastasis.

## Materials and methods

### Generation of genetically engineered KPC and KPCS mice

**Figure 5.**
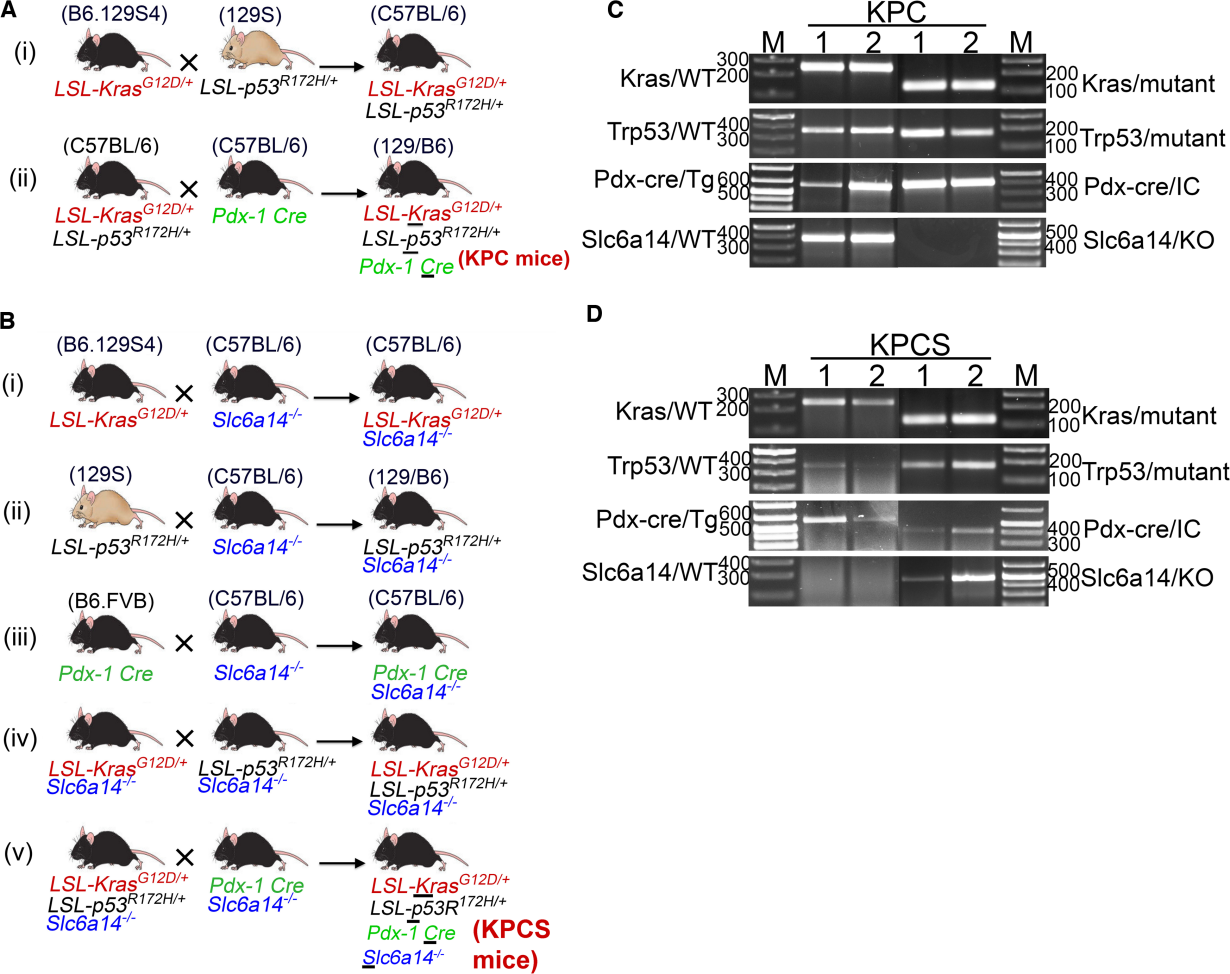
Generation of genetically engineered KPC and KPCS mice. Breeding strategy for generation of KPC mice using LSL-Kras^G12D/+^, LSL-p53^R172H/+^and Pdx1-Cre transgenic mice (**A**). Breeding strategy for generation of KPCS mice using LSL-Kras^G12D/+^, LSL-p53^R172H/+^and Pdx1-Cre transgenic mice and Slc6a14^−/−^ mice (**B**). RT-PCR showing representative KPC and KPCS genotype mice (**C**,**D**).

B6.129S4-Kras^tm4Ty^/J (also known as LSL-Kras^G12D^), 129S-Trp53^tm2Ty^/J (also known LSL-p53^R172H^), and B6.FVB-Tg(Pdx1-cre)6Tuv/J (also known as Pdx1-Cre) mice were all procured from The Jackson Laboratory (Bar Harbor, ME, U.S.A.). To generate the KPC spontaneous model of PDAC, LSL-Kras^G12D/+^ was first bred with LSL-p53^R172H/+^ to generate LSL-Kras^G12D/+^; LSL-p53^R172H/+^ (KP) transgenic mice. The KP mice were then bred with Pdx1-Cre mice to finally get LSL-Kras^G12D/+^; LSL-p53^R172H/+^; Pdx1-Cre (KPC) transgenic mice wherein mutated Kras and p53 are specifically activated in the pancreas due to the Cre recombinase driven by Pdx1 promoter (Figure 5A). Next, to generate KPCS mice, which is KPC mice in *Slc6a14*-knockout background, each of the transgenic mice i.e. LSL-Kras^G12D/+^, LSL-p53^R172H/+^, and Pdx1-Cre were individually crossed with *Slc6a14*^−/−^ mice to generate each of the transgenic mice in *Slc6a14*^−/−^ background (LSL-Kras^G12D/+^/Slc6a14^−/−^, LSL-p53^R172H/+^/Slc6a14^−/−^, and Pdx1-Cre/Slc6a14^−/−^). Thereafter, LSL-Kras^G12D/+^/Slc6a14^−/−^ mice were crossed with LSL-p53^R172H/+^/Slc6a14^−/−^ to get KPS (LSL-Kras^G12D/+^; LSL-p53^R172H/+^; Slc6a14^−/−^) mice. Finally, KPS mice were crossed with Pdx1-Cre/Slc6a14^−/−^ mice to get LSL-Kras^G12D/+^; LSL-p53^R172H/+^; Pdx1-Cre; Slc6a14^−/−^ (KPCS) mice (Figure 5B). PCR was performed to genotype the KPC and KPCS mice (Figure 5C,D). *Slc6a14*^−/−^ mice are available in house. All murine experiments were performed in the Laboratory Animal Resource Center at Texas Tech University Health Science Center in Lubbock, TX. The Texas Tech University Health Sciences Center Institutional Animal Care and Use Committee approved the animal experiments reported in the present study (IACUC protocol #18040).

### Mouse genotyping and real-time PCR

Genotyping was performed on each litter of mice as previously described [7]. In short, 5-mm tail snips were excised from mice, followed by lysis utilizing lysis buffer containing Proteinase K, DNA isolation, and PCR to identify mice that are positive for mutant Kras and p53, and positive for deletion of Slc6a14. The following PCR genotyping primers were used, as recommended by The Jackson Laboratory: for LSL-Kras^G12D^, wild-type forward: 5′-TGT CTT TCC CCA GCA CAG T-3′, mutant forward: 5′-GCA GGT CGA GGG ACC TAA TA-3′, and common reverse: 5′-CTG CAT AGT ACG CTA TAC CCT GT-3′. For LSL-Trp53^R172H^, wild-type forward: 5′-AGG TGT GGC TTC TGG CTT C-3′, mutant forward: 5′-CCA TGG CTT GAG TAA GTC TGC A-3′, and common reverse: 5′-GAA ACT TTT CAC AAG AAC CAG ATC A-3′. For Pdx-1 Cre, internal positive control forward: 5′-AGA TGG AGA AAG GAC TAG GCT ACA-3′, internal positive control reverse: 5′-CTG TCC CTG TAT GCC TCT GG-3′, transgene forward: 5′-CCT GGA CTA CAT CTT GAG TTG C-3′, and transgene reverse: 5′-AGG CAA ATT TTG GTG TAC GG-3′. *Slc6a14*^−/−^ mice were genotyped using the same protocol with the following primers: wild-type forward: 5′-AGC TCC TTT CTC AGC CTT CGG AAT-3′, wild-type reverse: 5′-TCC TTG TCA GCC AGT GAG GAA CAA-3′, *Slc6a14*^−/−^ forward: 5′-CAT GTT CTT ATT GGG CCT ACC-3′, and *Slc6a14*^−/−^ reverse: 5′-CCT GCC ATA GCC TCA GGT TAC TC-3′.

RNA isolation from mouse pancreas, cDNA synthesis, and qPCR were performed as previously described [7]. qPCR analysis was performed to analyze the expression profile of all known AATs in pancreatic tissues of *Slc6a14*^−/−^ mice as compared with *Slc6a14^+/+^* wild-type mice.

### Western blotting

Preparation of protein lysates from the tissues and the western blot analysis was performed as previously described [24]. In short, 20 µg of protein was loaded per lane in each of the western blots and electrophoretically separated on 10% SDS–PAGE gels. All proteins were transferred at 4°C overnight at 20 V to a nitrocellulose membrane (Bio-Rad), blocked with 5% Blotting Grade Blocker (Bio-Rad) at room temperature for 60 min, then incubated with primary antibody at 4°C overnight. Both primary antibodies (SLC6A14 Rabbit pAb, # AP16835 Abclonal; HSP60 Rabbit mAb #12165 Cell Signaling) were diluted 1000-fold. After washing with TBST, the membrane was incubated for 1 h at room temperature with goat anti-rabbit secondary antibody (Bio-Rad) at a dilution of 3000-fold, washed in TBST again, then incubated for 5 min in Pierce ECL solution (ThermoFisher), before being visualized with autoradiography films.

### Tissue preparation and histology

KPC and KPCS mice were monitored on a regular basis to check for symptoms of abdominal distension and cachexia following which they were killed via isoflurane induction. Tissues were then harvested following the approved IACUC protocol. Gross examination was performed post-mortem to check for the presence of hemorrhagic ascites, primary tumor, and metastatic foci in the surrounding tissues like the liver, diaphragm, stomach, intestine, spleen, kidneys, and the lungs. The pancreas and the associated tissues were immediately fixed in 10% neutral buffered formalin for histopathology. The TTUHSC Department of Pathology performed all histopathological analyses on the pancreas and the associated tissues to determine the histological diagnosis and the presence of metastasis (Figure 3A,B).

### Immunohistochemistry

Immunohistochemistry studies were performed as previously described [5]. In short, KPC and KPCS pancreatic tumor sections were cleared with xylene, rehydrated, and stained according to the Vectastain ABC Kit Protocol (Vector Labs, PK4000). The tissue sections were then stained with primary antibodies like anti-Ki67 1 : 200 (Cell Signaling, D3B5), anti-α-SMA 1 : 100 (Abcam, ab5694), or anti-CK19 1 : 400 (Abcam, ab52625). Tumor samples were then stained with secondary antibody 1 : 200 (Vector, BA-1000), followed by washing, DAB color development, hematoxylin staining, and mounting with ImmunoHistoMount (Sigma, I1161). All images were captured using a Nikon Eclipse Ts2 microscope with NIS-Elements software.

### Statistics

GraphPad Prism 8.4.3 was used to generate the Kaplan–Meier curve for survival analysis. Statistical significance was determined by the log-rank (Mantel–Cox) test and the unpaired Student's *t*-test. Results are expressed as mean ± SEM.

## Data Availability

All datasets generated and/or analyzed during the current study are included in this manuscript.

## Open Access

Open access for this article was enabled by the participation of Texas Tech University Health Sciences Center in an all-inclusive *Read & Publish* agreement with Portland Press and the Biochemical Society.

## Abbreviations

α-MLT: α-methyl-l-tryptophan
α-SMA: alpha-smooth muscle actin
AAT: amino acid transporter
CK19: cytokeratin 19
ER: estrogen receptor
MMPs: matrix metalloproteinases
PDAC: pancreatic ductal adenocarcinoma
PSCs: pancreatic stellate cells
